# Reply To—Gender Distribution Among Transplant Journal Editorial Members

**DOI:** 10.3389/ti.2022.10262

**Published:** 2022-03-17

**Authors:** Deborah Verran, Annemarie Weissenbacher, David Paredes-Zapata, Fernanda Ortiz

**Affiliations:** ^1^ Ramsay Health Care, Sydney, NSW, Australia; ^2^ Department of Visceral, Transplant and Thoracic Surgery, Center of Operative Medicine, Medical University of Innsbruck, Innsbruck, Austria; ^3^ Donation and Coordination Section, Hospital Clinic, Department of Surgery and Surgical Specializations, Faculty of Medicine, University of Barcelona, Barcelona, Spain; ^4^ Abdominal Center Unit, Nephrology, Helsinki University Hospital, Helsinki, Finland

**Keywords:** gender, equity, transplant, journals, editors, data

Dear Editors,

We read with interest the recent publication in Transplant International by Lim et al. (1) on the gender ratios of professionals on the editorial boards of transplant scientific journals. The authors provide summary data for the ratios of men versus women for the editorial boards plus associate editors positions of 29 journals sourced via Scimago. They demonstrated that there is a disparity in the gender ratios for these particular positions. No other specific information is provided about these particular journals bar one.

This is an important issue because it has become evident that there is a degree of gender disparity for all of the listed editorial-type positions across a range of top medical and surgical journals published around the world (2–4).

We wonder whether Lim et al may have analysed website information for both cellular and solid organ transplantation journals (1). By examining the websites of the 45 “transplant” journals currently listed via Scimago, we discovered that 10 were no longer being published, 6 were not published in English, 4 had a focus on cellular therapies only, 2 were directed at a non-medical audience and 1 had the Chief Editor only listed. This left us with 22 potential solid organ transplant journals in comparison to the 29 found by Lim et al. This raises the question of whether there has been some inadvertent introduction of additional variance into their results due to a lack of uniformity of the journals that they selected.

It is now known that there is a reasonable amount of variation between medical and surgical journals as to how many females are either associate editors or chief editors, which ranges in reports from 0 to 82% (2–6), with concern being expressed over the lack of gender equity for the top tier positions. With the summary data as reported by Lim et al being broken down into quartiles this does not allow for any further understanding to be gained by the reader as to where the variance exactly lies between all of the transplant journals for the full range of listed editorial positions.

A preliminary analysis of the Chief Editors of the 22 journals we located revealed that 4/22 (18%) are female compared to 32.3% of the associate editors of the 29 journals obtained by Lim et al. There is also a range of second tier editorial positions listed for transplant journals including Deputy editors, associate editors, editors and scientific editors, which are potentially the pool of individuals all designated as associate editors by Lim et al. The discrimination of the gender distribution in a full range of editorial positions we identified is shown in Figure 1. Noteworthy, 7 out of 22 journals (32%) do not have women in the top tier positions along with the 1 journal with all-men in the editorial board.

**FIGURE 1:**
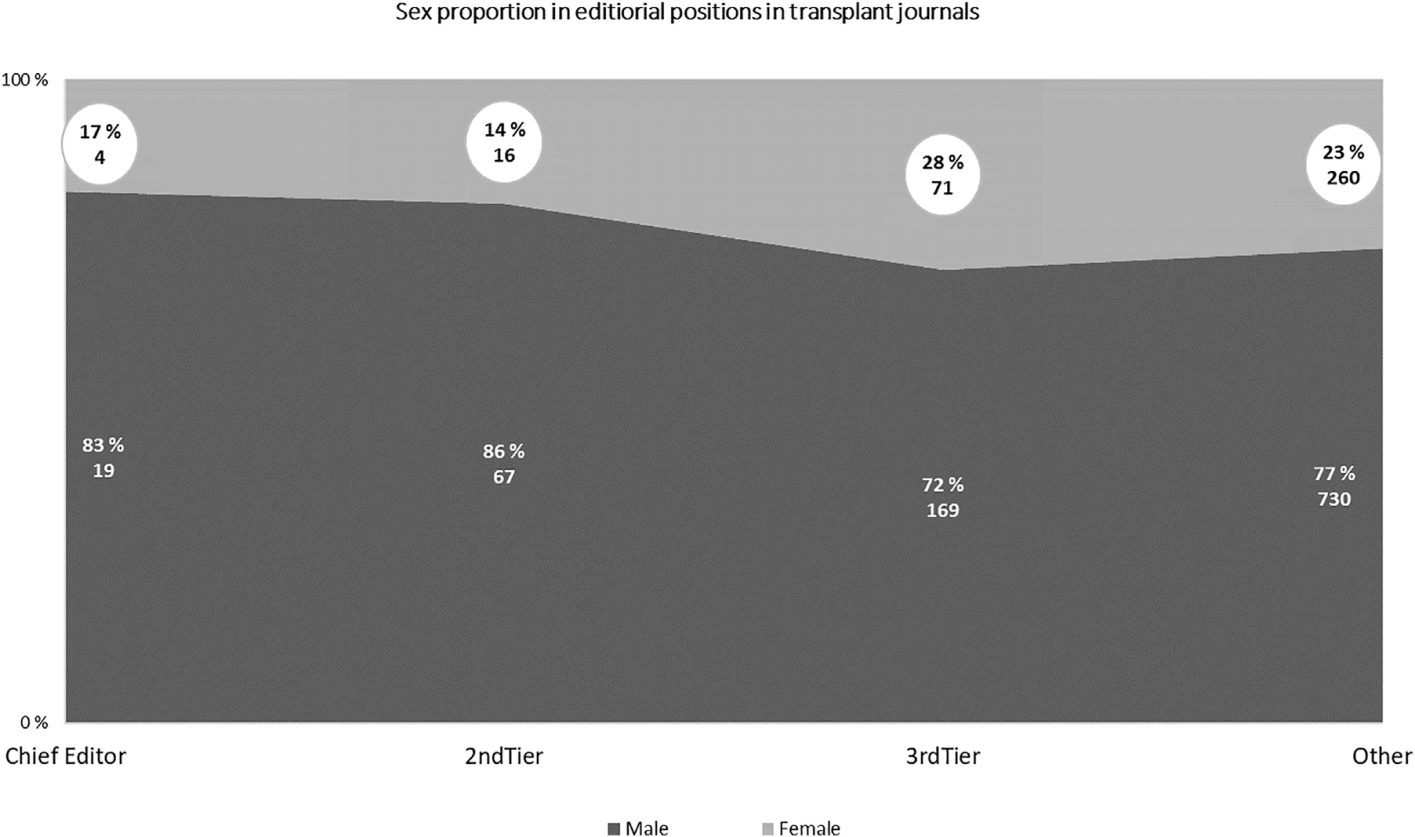
Number and percentage of female representation in 22 transplant journals (Transplantation, Am J Transpl, Neph Dial Transpl, Liver Transplantation, JHLT, Trans Proc, Transpl Int, Clinical Transpl, Paediatric Transpl, Transpl Infect Disease, Transpl Immunology, Xenotransplantation, Current Opin Organ Transpl, Transplantation Reviews, Annals of Transpl, Saudi J Kid Disease Transpl, Experimental Clin Transpl, Transpl Direct, Transpl Research Risk Management, Turkish Nephrology Dialysis Transpl J, Transplantation Reports, Cell and organ Transplantology).

Nevertheless, we agree with Lim et al. that there are discrepancies in the gender ratios which have implications for corrective actions (7), noting that some transplant journals are already adopting specific measures (8).

## Data Availability

The raw data supporting the conclusion of this article will be made available by the authors, without undue reservation.
